# Knowledge‐based representation: Patient engagement in drug development

**DOI:** 10.1111/hex.13912

**Published:** 2023-12-22

**Authors:** Claudia Egher, Olga Zvonareva

**Affiliations:** ^1^ Innovation Studies, Copernicus Institute of Sustainable Development Utrecht University Utrecht The Netherlands; ^2^ Department of Health, Ethics and Society, Faculty of Health, Medicine and Life Sciences Maastricht University Maastricht The Netherlands

**Keywords:** drug development, knowledge, knowledge‐based representation, participation, patient engagement, representation

## Abstract

**Introduction:**

Recently, different actors have intensified their efforts to make drug development more participatory. They have produced many frameworks, tools and dedicated fora, where patients are portrayed as relevant stakeholders to be involved throughout the entire drug development trajectory. To better understand what such participatory efforts entail, in this article, we investigate how patient representation is configured in drug development and what patients can engage as representatives in this field.

**Methods:**

This is a qualitative study based on the thematic analysis of 40 semistructured interviews with different stakeholders in the field and three patient engagement How‐To guides (HTGs) complemented by observations of two sessions of the Patient Engagement Open Forum (PEOF) and a patient expert training of the European Patients' Academy on Therapeutic Innovation (EUPATI).

**Findings:**

The emerging practices of patient engagement in drug development configure representation as hinging upon three types of knowledge—drug development knowledge, autobiographical knowledge and community knowledge—and a specific set of skills. We discern a new kind of representation based on these findings, termed ‘knowledge‐based representation’, which appears to more accurately describe how patients are expected to represent others in drug development.

**Conclusion:**

Even though knowledge‐based representation may be understood as an attempt to downplay the political aspects of representation in favour of its epistemic elements, the political processes involved in patient representation in drug development cannot be ignored. The extent to which reliance on knowledge‐based representation will contribute to democratic decision‐making is likely to depend on the resources needed to develop the types of knowledge relevant to representation work and on how these types of knowledge are determined.

**Patient or Public Contribution:**

Patient representatives and practitioners in the field of patient engagement (including 13 interviewees, representatives of EUPATI and HTG developers) gave feedback on the interpretation of the findings during a multistakeholder workshop we organised. We also sent an interviewee an extended draft and discussed it during an online meeting. Claudia Egher presented these findings at a PEOF session in June 2023, which further contributed to their validation.

## 1 INTRODUCTION

A participatory turn is taking place in drug development. In contrast to health care, where participation has been promoted for decades,^1^ drug development has remained largely insulated from this trend until the 2010s,^2^ when calls and efforts ‘to ensure that patients and their needs are embedded at the heart of medicines development and lifecycle management’^3^ ^,p.929^ emerged. By now numerous frameworks, tools and events have been put forward to encourage patient engagement (PE) in drug development.^4^

The efforts to further PE in drug development have been undertaken by regulators, pharmaceutical companies, patient organisations and multistakeholder groups. In 2012, the US Food and Drug Administration launched the Patient‐Focused Drug Development initiative,^5^, ^6^, ^7^ followed in 2016 by the European Medicines Agency (EMA) that set up a ‘cluster’ on PE in drug development.^8^ The pharmaceutical industry has contributed by developing PE tools, such as the Patient Protocol Engagement Toolkit created by TransCelerate Biopharma Inc., and by providing (financial) support to multistakeholder groups dedicated to PE.^9^ One example of the latter is the global not‐for‐profit initiative Patient‐Focused Medicines Development (PFMD) established in 2015 and central to the analysis in this paper. PFMD has developed numerous PE tools and training and co‐hosted dedicated events.

Among other ways, patient organisations have participated in furthering PE in drug development by training patients. For example, in 2012, the European Patients' Forum (EPF), an umbrella patient organisation, supported the establishment of the European Patients' Academy on Therapeutic Innovation (EUPATI). EUPATI provides education ‘to increase the capacity and capability of patients and patient representatives to understand and meaningfully contribute to medicines research and development’.^10^ Together with EPF and PFMD, in 2019, EUPATI launched the Patient Engagement Open Forum (PEOF). This series of regular events is dedicated to sharing new PE tools, discussing challenges and facilitating collaboration. PEOF has become central to the contemporary landscape of PE in drug development.

### 1.1 Aims

With the rise of enthusiasm for PE in drug development, it is important to examine how PE is being configured. Particularly, PE entails a few engaged patients speaking on behalf of or, in other words, representing, many others. Current scholarship and the emerging practice of PE in drug development tend to assume that such representation is unproblematic. Yet, political science and science and technology studies (STS) scholarship suggest that representation cannot be taken for granted, as it is always an uncertain, incomplete and ongoing accomplishment.^11^, ^12^, ^13^ Against this background, this paper aims to (1) gain insight into who can become patient representatives and (2) explore the implications of how representation is conceived for shaping PE in drug development.

### 1.2 Theoretical framework: Representation and its challenges

The so‐called ‘standard approach’ to representation conceives of representation as a dyadic, unidirectional principal–agent relationship, whereby a unified group elects representatives to act on its behalf.^11^, ^14^, ^15^ The representatives are expected to have knowledge about their constituencies' interests and to adopt policies that correspond to them, that is, to behave as their constituencies would.^16^ This perspective embraces a realist take on the interests of a constituency, as it assumes that they are available and stable. Furthermore, these interests are assumed to develop according to general demographic factors, such as age, gender and ethnicity.

The ‘standard approach’ has been challenged by political scholars of a constructivist bent,^13^, ^17^, ^18^, ^19^ who conceive of representation as ‘a dynamic process that mobilizes and shapes constituencies in a wide range of venues’.^11^ ^,p.2^ Constructivist scholars have argued that preferences cannot be directly deduced from general demographic factors or shared experiences. Instead, preferences or social perspectives are shaped by an individual's life trajectory, the multiple groups s/he belongs to and the process of political mobilisation itself. As the constituencies' preferences are understood to co‐emerge in the process of representation, individuals become representatives through their direct experiences and efforts to articulate an issue.

This paper draws upon the constructivist approach to analyse how patient representation is configured in drug development and who can act as representatives. Specifically, it draws on the political science understanding of representation as relying on the ability to make representative claims^20^ and on STS insights that representative claims are epistemic, with knowledge ascribed a central role in efforts to shape matters of collective concern.^21^, ^22^, ^23^

## 2 METHODS

### 2.1 Study design

This is a qualitative study underpinned theoretically by a constructivist approach to representation. It relied on three avenues of data collection: semistructured interviews, document analysis and participant observations. Going beyond a single type of data was necessitated by the emerging and under‐researched character of our field of study as well as by the complex and elusive nature of representation. Bringing together interviews, documents and observations allowed us to critically compare perspectives on representation and, thereby, elicit often tacit expectations regarding what makes for a good patient representative in the field of drug development. Interview‐ and document‐based data were analysed using thematic content analysis, while observation data were used to enrich and better contextualise the resulting analysis. The study was designed to be sensitive to the insights emerging in the process of research and to ensure adaptation to them by including additional participants and topics to explore. This in‐built flexibility^24^ allowed reaching depth and comprehensiveness in characterising representation.

### 2.2 Data collection and participants

Since PE in drug development is a field shaped by many different actors as delineated in the Introduction, our data collection strategy focused on PFMD and EUPATI as multistakeholder groups where regulators, pharmaceutical companies and patient organisations collaborate. Such focus allowed bringing these diverse actors together analytically as stakeholders. PFMD and EUPATI were selected due to their prominence in terms of the volume of PE initiatives and intensity of collaborations.

The first avenue of data collection was semistructured interviews. Claudia Egher conducted 40 semistructured interviews with patient experts, patient advocates, patient education providers, members of the pharmaceutical industry and regulators, many of whom combined different roles from this list (for details see Table 1). One repeat interview was conducted with a member of the EUPATI management team, with the two interviews focusing on the participant's two different roles. In several instances, more time was needed to complete the interviews than initially allocated, so another session was planned to complete it. Such sessions are indicated in Table 1 through the letter ‘C’ added to the interview number and together with initial interviews were counted as one.

**Table 1:** Overview of interviewees.

Interview No.	Gender	Function
I1	Female	Management position EPF
I2	Female	Former pharmaceutical industry professional; consultant
I3C	Female	Former pharmaceutical industry professional; consultant
I4	Female	Pharmaceutical industry professional
I5	Male	Pharmaceutical industry professional
I6	Female	PFMD representative
I7	Female	Patient advocate, PE champion, consultant
I8	Male	Consultant; HTG co‐developer
I9	Female	Former pharmaceutical industry professional; consultant; PE trainer
I10	Female	EUPATI representative
I11	Female	Pharmaceutical industry professional
I12	Female	Pharmaceutical industry professional, patient, HTG co‐developer
I13	Male	Pharmaceutical industry professional; PFMD representative; HTG co‐developer
I14	Female	European Infrastructure for Translational Medicine representative
I15C	Female	Pharmaceutical industry professional; patient; HTG co‐developer
I16	Female	Patient representative/ambassador; patient
I17	Female	Pharmaceutical industry professional; HTG co‐developer
I18C	Female	European Infrastructure for Translational Medicine representative
I19	Male	EUPATI patient expert; patient advocate; patient
I20	Female	PE expert at a company focusing on PE; former manager of patient organisation
I21	Male	EUPATI patient expert; member/founder of multiple patient organisations; patient
I22	Female	Patient advocate working at umbrella patient organisation
I23	Female	Patient advocate; patient; EUPATI patient expert
I24	Female	EUPATI patient expert; consultant; patient
I25	Female	PFMD representative; PE research coordinator
I26	Female	Former pharmaceutical industry professional; manager of a patient organisation; PFMD representative
I27	Female	Pharmaceutical industry professional
I28C	Female	Former pharmaceutical industry professional; manager of a patient organisation; PFMD representative
I29	Female	Academic researcher; patient; HTG co‐developer
I30	Female	EUPATI patient expert; carer
I31	Male	EUPATI patient expert; patient
I32	Female	Academic researcher; EUPATI patient expert; patient
I33	Female	EUPATI patient expert; former carer
I34	Female	EUPATI representative (2)
I35	Female	Member of regulatory agency
I36	Female	EUPATI representative and trainer; EUPATI patient expert; patient
I37	Female	EUPATI trainer; member of regulatory agency; academic
I38	Male	EUPATI trainer; patient
I39	Male	EUPATI patient expert; patient
I40	Female	EUPATI patient expert; patient
I41	Female	EUPATI patient expert; patient
I42	Female	EUPATI representative; carer
I43	Male	EUPATI trainer; member of regulatory agency
I44	Male	EUPATI patient expert; consultant; patient

Abbreviation: EPF, European Patients' Forum.

To recruit participants for an interview, purposeful and snowball sampling were used. Members of PFMD and PFMD collaborators were first invited since PFMD is both a key international player in the field and a highly heterogeneous group in terms of actors involved. Snowball sampling was subsequently used, as the participants were asked to recommend other potential interviewees. Since PE in drug development is a relatively new field and not all information about key initiatives in the making is publicly available, snowball sampling allowed us to ensure that we did not overlook relevant participants. Importantly, since the initial interviews established the central relevance of EUPATI, EUPATI representatives were also contacted. These participants came mainly from European countries, with two participants from the United States, one from Australia and one from Zimbabwe.

An interview guide was developed with a focus on topics, such as the interviewees' understanding of PE and the role of patient education for participation in drug development; who was included/excluded from PE in drug development and how; how the interviewees engaged in PE and why; who and what they represented during PE activities; the quality of their interactions with other stakeholders and so forth. During the interviews, the participants were encouraged to also share insights they found relevant even if these were not included in the guide. Most interviews were conducted online (35), with five conducted in person during the EUPATI training. During both online and offline interviews, only Claudia Egher and the interviewee were present. All interviews were audio‐recorded with the consent of the participants. The duration of the interviews varied between 45 and 180 min. To acknowledge interview participation as a form of labour, the interviewees whose direct quotes are provided in this article were allowed to decide on the level of anonymity they preferred upon reading the full manuscript. As a result, a combination of real names and pseudonyms is used, to respect both the wishes of the interviewees who wanted their contribution to be publicly acknowledged and of those who opted for anonymity.

The second avenue of data collection was the collection of documents. The documents scrutinised were three How‐To Guides (HTGs) published and promoted by PFMD between 2019 and 2021: *How‐to Guide for Patient Engagement in the Early Discovery and Preclinical Phases*; *How‐to Guide on Patient Engagement in Clinical Trial Protocol Design*; *Plain language summaries (PLS) of Peer‐Reviewed Publications and Conference Presentations: Practical ‘How‐To’ Guide for Multi‐stakeholder Co‐creation*. These documents were selected because their purpose was to shape PE during different phases of drug development and they were developed collaboratively by heterogenous groups of stakeholders.

The third avenue of data collection was participant observation. Claudia Egher observed two online PEOF sessions (2022) and the EUPATI Patient Expert Training in Madrid (2022). A list of elements to focus on during the observations was developed based on insights from the literature and the interviews already conducted. This list was adjusted during the process of observation, to better fit the content and behaviours observed and to allow for the inclusion of unexpected relevant insights. Extensive field notes were taken during the observations. They contained Egher's impressions and descriptions of the participants' gestures and nonverbal behaviours as well as of the slides they used.

### 2.3 Data analysis and rigour

Data analysis started soon after the commencement of data collection and subsequently, the two proceeded simultaneously. Interview‐ and document‐based data were analysed through thematic content analysis.^25^ While the data were still being collected, the analysis proceded in the following way: we identified prominent themes in the data, we wrote analytical memos, and we discussed the themes and memos among the two of us as well as with the wider research group. In the process, patterns of meaning were delineated and critically assessed and additional questions requiring exploration were formulated and fed back into the data collection process. Multiple such iterations took place until data saturation was reached, when limited new relevant insights, issues and topics were gained from new interviews.

Further, the coding was conducted using the software Atlas.ti. It started with the careful reading of the interviews and documents to allow immersion in the data. Claudia Egher then open‐coded the transcripts of the first interviews, the results of which and the emerging coding scheme were discussed with Olga Zvonareva. The insights from this discussion informed the subsequent round of open coding by Egher. Early in this iterative process, the prominence of representation was revealed together with its close connection to knowledge. From this point, the coding focused on how representation and patient representatives were constructed in drug development and what this meant for shaping PE in the field. The final coding scheme was developed both deductively and inductively, being informed by the literature on representation and derived from the data. Definitions of and interrelations between all themes and subthemes were discussed and agreed upon by the authors. An overview of the coding scheme can be found in Table A1. Parts of interview transcripts and documents corresponding to different codes were examined by both authors together, to ensure agreement in interpretation. In several instances of disagreement, reasons for the (in)applicability of a certain code were discussed, invariably leading to an agreement. When the coding was completed, the fieldnotes taken by Egher during the observations were read and juxtaposed with the coded interviews and documents. The insights from all three data types were placed in a dialogue with each other, allowing for more nuance and depth, and, ultimately, for triangulation, thus ensuring the robustness of the analysis.^26^

The findings were shared with the research participants and broader communities of stakeholders during a workshop on PE in drug development^27^ the authors organised in 2022 (Maastricht University, 2022) (13 interviewees were among the attendees) and during a dedicated PEOF session in 2023. The participants in both events recognised and agreed with the findings and shared additional experiences in support of the analysis. Furthermore, during the PEOF co‐creation session, Egher reflected together with relevant stakeholders on how the issues regarding patient representation in drug development identified in this paper could be addressed.

## 3 FINDINGS

Our analysis showed that patients are expected to possess three different types of knowledge—drug development knowledge, autobiographical knowledge, community knowledge—and what we call ‘adaptive skills’, to perform diverse representation work in drug development.

### 3.1 Drug development knowledge

Our findings revealed a shared expectation that patients need to be endowed with drug development knowledge to fruitfully contribute to new medications. The expected drug development knowledge consists of: a basic understanding of the drug development stages and familiarity with scientific terminology; insights into the organisation and practices of pharmaceutical companies; awareness of the main rules and regulations shaping the drug development process and the inner functioning of regulatory bodies. Patients were expected to be capable of engaging in different types of representation work depending on their level of proficiency.

First, most interviewees expected patients with a basic level of drug development knowledge to be able to give feedback on and/or co‐write materials, such as informed consent forms, clinical trial protocols and plain language summaries. Patients were thought to acquire this level of drug development knowledge through self‐study, participation in clinical trials or short training, as the following quote by a patient representative illustrates:

Probably for the first year and a half, I just listened and didn't really know what was going on, or really understood the small parts of it. But gradually, as new trials were launched, the sponsors of those trials would get in touch with me, give me an explanation of it, and ask me if I would come on board. (…) So, I feel more involved now and I understand more. I'm able to contribute and give the patient's view on things … and do a lot of reviewing of lay summaries of the patient information sheets that they're given at the start of the study, just to review. (Helen Carter)

Second, patients with more substantial drug development knowledge were expected to represent others by giving feedback to researchers on study designs, to ensure the latter's alignment with patient preferences regarding the outcomes assessed and the indicators employed to measure them. The following quote by a patient education provider illustrates the importance accorded to substantial drug development knowledge:

I could see that the HIV movement has really produced such knowledgeable patient advocates who are able to step into conversations with researchers … These conversations are about clinical trials and really detailed information about how treatments are developed. I see that the power of patient advocacy at its best is when a person is informed, trained, and able to speak at the same level as the researcher. (Maria Dutarte)

Achieving this level of drug development proficiency was, however, thought to require specialised and intensive training.

Third, patients endowed with in‐depth drug development knowledge were expected to represent others by contributing to regulatory activities, as the following quote from a research organisation employee recounting an exchange about patients joining EMA committees, suggests:

And I remember telling that to someone working at *Y* [a patient education provider], and he told me like, ‘Yeah, you know, we train 30 people a year, but we really only have one or two people per edition who actually can do this type of work, who actually have the shoulders, and the knowledge, and the understanding to even sit on those committees’. (Mette Grimsdottir)

A few interviewees doubted that substantial drug development knowledge was always necessary for patients to represent others in drug development:

This is another discussion we had with *W* [another patient education provider] and some patient groups who insisted that only experienced ‘patient experts’, as they call it, should give input to pharma. When I was still at *Z* [pharmaceutical company], we made an inventory of the types of questions and asks we had from patients (…) And we found that for about 85% of all our demands, all you needed was to have the disease. The less you are biased by knowing how research works, the better. When it's for regulatory documents, like leaflets or submission … or basic research, then it is truly good to also have some patient experts like *W* graduates. But for the vast majority, that's not the case. (Pablo Nereira)

However, most interviewees considered substantial drug development knowledge necessary for patients to perform representation work.

### 3.2 Autobiographical knowledge

Another important theme was the expectation for patient representatives in the field of drug development to be endowed with autobiographical knowledge. This is the knowledge that individual patients develop through their embodied experiences. It encompasses perspectives and insights through which a condition is made sense of at the level of one's entire life, based on one's values and preferences. Thus, autobiographical knowledge does not refer to the symptoms one experiences, or to the (side‐)effects of medications in isolation, but to how they shape one's quality and trajectory of life.

Autobiographical knowledge was framed as unavailable to nonpatients and was used to highlight the insufficiency of insights that could be acquired from other types of representatives, as the following quote by a pharmaceutical company professional illustrates:

If you are talking about two symptoms, one symptom is pain. The HCPs [health care professionals] can never reflect the pain the patient feels, because they are not feeling the pain. Another symptom is itch. How do you describe itch? I used to work on the skin conditions and skin health. No, there is no way to describe itch by HCPs. Only patients using the analog or some electronic scales can reflect that. But doctors can never describe such symptoms. (Oleks Gorbenko)

Thus, part of the representation work that people endowed with autobiographical knowledge were expected to do was to (help) develop a language through which personal experiential states could be translated and transferred to those lacking this knowledge. In ways that echo realist approaches to representation, this expectation relies upon the assumption that bodily states would be similarly experienced by different patients.

Autobiographical knowledge was further expected to enable patients to perform representation work by assisting researchers in the early stages of drug development, by providing thick descriptions of their lives with a condition and by making sense of the priorities and preferences the researchers identified using quantitative approaches.

Some interviewees believed, however, that autobiographical knowledge was individualised and lacked the level of commonality required to represent others, as the following quote illustrates:

What I've learned is that every patient has a very different experience. Even if the patients are diagnosed with the same condition and follow similar treatment paths, there will always be differences; there will always be different understandings and different views on things. So, I can't represent all *A* [medical condition] patients; I can only talk about my experience and my feelings. I can only really represent myself. (Helen Carter)

Nevertheless, most interviewees and training participants viewed autobiographical knowledge as an important resource through which interests, perspectives or insights relevant to more than one person could be mobilised and acted upon. This view entails that autobiographical knowledge involves not only a reflection on one's experiences but also validation through interactions with other patients.

### 3.3 Community knowledge

Patient representatives were also expected to have community knowledge, which denotes insights about how a broad group of patients are affected by specific ill‐health conditions. It was expected to be developed by people with autobiographical knowledge about a condition and those without. Even though autobiographical knowledge was thought to increase the legitimacy of the representative claims made by patient representatives endowed with community knowledge, community knowledge was expected to mitigate the risk that patient representatives might represent their own interests rather than those of broad groups. The following quote from a patient organisation staff member and education provider illustrates this: ‘it is part of our mission to make sure that advocates have the kind of knowledge they need to think beyond their own personal experience’ (Vivian Guillaumes). Community knowledge may thus be construed as both an epistemic and normative resource, ensuring the availability of knowledge about a community and adequate representation. Interestingly, we found no instances in our data where checks and balances regarding patient representation in drug development were openly discussed.

Community knowledge was considered more time‐sensitive than the types of knowledge discussed above, requiring frequent updates to remain relevant for representation work. It could be acquired by immersing oneself in already available data or by engaging in data collection in quantitative and qualitative ways. Whereas the collection and analysis of quantitative data were expected to lead to the construction of hierarchies of needs and preferences, acquiring community knowledge in qualitative ways was expected to ensure the latter's validity and relevance.

Overall, community knowledge was expected to ensure a high level of correspondence between the hierarchy of needs and preferences put forward by the patients engaging in representation work and the experiences of their communities, as the following quote suggests:

You need to understand your patients really well. (…) You can be versed in the research and hearing things through that by attending congresses and things, but you need to spend a little bit of time on the ground, so to speak, in order to really maintain that connection, and [so] that the information that you are providing is representative. (Anja Duval)

Furthermore, community knowledge was expected to counteract the alienating effects that reaching a high level of proficiency in drug development knowledge might bring about between representatives and the groups they are meant to represent.

### 3.4 Adaptive skills

The three delineated types of knowledge were expected to be successfully used in patient representation in drug development, provided that the patient representatives were also endowed with what we call ‘adaptive skills’. Adaptive skills function as a vehicle through which patients can perform representation work, as they help ensure that any type of representation work is found persuasive and legitimate. Adaptive skills denote capabilities that enable one to adjust to other stakeholders and environments and to make normative as well as pragmatic decisions to ensure the successful completion of one's representation work, as the following quote suggests:

That's really important. You can know all about the phases of drug development, but when you're not able to work together as a team and also … look from the side of the other stakeholders, then you … cannot come to a compromise. (Alena Sapoznikov)

The interviewees and the forum participants connected the patients' ability to represent others to their level of communication skills and practical experience. For example, a speaker at a PEOF session classified different levels of patient preparedness for engagement and, correspondingly, for representing others in the following way (Table 2).

Adaptive skills were thought to enable patients to correctly assess different participatory circumstances and to render them capable to resist situations marked by power imbalances, where they were expected to ‘go, see, and shut up’ (Toni Montserrat). At the same time, this set of skills was thought to enable patients to focus on what they could gain in less‐than‐ideal situations. For instance, the trainees were advised that: ‘Even if you don't like it, deal with the system in front of you, not with the one you'd want it to be. The latter you can pursue some other time, in other ways’ (Participant Observation Notes‐271022). Furthermore, adaptive skills were also expected to allow patients to handle interactions that might challenge their own views and interests.

**Table 2:** Tiering expertise.

Tiering expertise			
Global	Tier 1	Tier 2	Tier 3
Experience with product or disease state	Experience with product or disease state	Experience with product or disease state	Engagement solely based on experience with product or disease state
Demonstrated ability to speak and/or to share	Little‐to‐no‐ related public speaking experience	Established but less influential presence	Typically does not engage in public speaking and/or influencing
Broad reach of the audience	Limited reach of the audience	Limited reach of the local audience	

*Note*: Developed based on a presentation titled ‘Fair Market Value and PE’ given by a speaker at a PEOF session on 23 February 2022.

## 4 DISCUSSION

In the context of the ongoing participatory turn in drug development, this study scrutinised how representation is configured in this field. Our findings show that patient representation in drug development is conceived as requiring drug development knowledge, autobiographical knowledge, community knowledge and adaptive skills. Variations in these types of knowledge and skills are linked to differences in the complexity of the representation work that patients are expected to be able to perform.

Our findings point towards the emergence of a new kind of representation—knowledge‐based representation. Knowledge‐based representation denotes a process whereby representatives emerge as a result of the development of relevant epistemic resources that serve to legitimise the work they do on behalf of others. Thus, while it still focuses on one's ability to articulate representative claims, as in the work of constructivist scholars of representation,^13^, ^28^, ^29^ knowledge‐based representation foregrounds the types of knowledge that are needed for different claims to be considered representative.

The notion of knowledge‐based representation also differs from realist conceptualisations. First, representatives are not thought of as having access to stable and already available interests but are expected to engage in representation work based on domain‐specific and varied types of knowledge that are viewed as necessary, even when the representatives and the represented share specific concerns and experiences. Second, representation is not understood as a monolithic endeavour consisting of similar actions. Instead, representation is conceived of as a diverse set of activities and behaviours of varying complexity and significance. Third, knowledge‐based representation implies a more complex web of relationships than the unidirectional ones of the ‘standard approach’,^14^ as patient representatives are expected to establish and maintain connections with diverse stakeholder groups at different moments and levels of their representation work in drug development.

Reliance on a knowledge‐based conception of representation has practical implications. For example, insistence on substantial levels of drug development knowledge may limit the diversity of patients who can perform representation work in drug development, as only those with enough free time, financial resources and determination to acquire such knowledge may become suitable candidates for engagement. The expectation of considerable adaptive skills may facilitate the formation of a small group of repeat participants in PE activities, as only a few patients might satisfy this expectation due to the significant amount of practice needed and these few patients would consequently be approached by multiple organisers. Thus, even though knowledge‐based representation may be understood as an attempt to downplay the political aspects of representation in favour of its epistemic elements, the political processes involved in patient representation in drug development cannot be ignored. Whether or not this new type of representation will afford more democratic decision‐making processes and under which conditions remains an open and intriguing question.

### 4.1 Limitations and recommendations

Our study is confined to materials conveying recommendations and expectations. Future ethnographic studies are needed to understand how the expectations discussed here are enacted in different settings and at different stages in the drug development trajectory. As the materials we analysed have a European focus, additional research is needed to understand how patient representation in drug development is configured in other geographic areas. The findings of this study indicate that who can represent patients in drug development matters and point to the risk of the development of a narrow patient representative elite. We, therefore, recommend practitioners interested in ensuring the democratic character of patient representation in drug development to pay closer attention to the actors and methods involved in determining the types of knowledge needed for different representation work. Efforts should be made to facilitate the development of the types of knowledge relevant to representation in drug development among diverse patient groups. Furthermore, we recommend the development of a system of checks and balances to ensure the quality of patient representation in this field.

## AUTHOR CONTRIBUTIONS

Both authors contributed to the conceptualization of this article, to its writing, reviewing, and editing. Claudia Egher collected the data. Both authors approve of this document.

## CONFLICT OF INTEREST STATEMENT

The authors declare no conflict of interest.

## ETHICS STATEMENT

Our study was approved by the Ethics Review Committee of the Faculty of Health, Medicine and Life Sciences of Maastricht University (FHML‐REC/2021/108).

## Data Availability

The data that support the findings of this study are available on request from the corresponding author. The data are not publicly available due to privacy or ethical restrictions.

## APPENDIX A

### A.1

**Table A1:** Overview of the main coding scheme.

Theme	Category	Code
Drug development knowledge	Components of drug development knowledge	Knowledge about drug development stages
		Knowledge about scientific terminology
		Knowledge about the functioning of pharmaceutical companies
	Levels of drug development knowledge	Basic
		Moderate
		High
	How drug development knowledge can be acquired	Self‐learning/individual information practices
		Basic training/educational activities
		Complex training/intense, time‐consuming educational activities
	Representation activities based on drug development knowledge	Giving feedback/reviewing clinical trial materials
		Suggesting how questions and explanations for patients should be phrased in materials to be used by drug developer teams in their interactions with patients
		Suggesting content for clinical trials and other materials used by drug developer teams
		Co‐writing materials to be used by drug developer teams in their interactions with patients
	Representation‐relevant risks	Small pool of patients
		Co‐optation
		Limited originality of suggestions
Autobiographical knowledge	Components of autobiographical knowledge	Insights about the symptoms and manifestations of one's condition
		Capacity to indicate which symptoms and manifestations one finds more or less tolerable and why
		Insights about the effects of one's treatment
		Capacity to indicate which treatment effects one finds most important
		Insights about the side effects of one's treatment
		Capacity to indicate which treatment side effects one finds most serious/disturbing
		Insights about treatment needs/improvements one would like to have
	Level of autobiographical knowledge	Limited experience with a condition due to a recent diagnosis
		Extensive experience with a condition due to many years of having been diagnosed with it
	Representation activities based on autobiographical knowledge	To describe in detail one's experiences with a condition that is not sufficiently addressed through available treatments
		To describe in detail one's treatment needs that are not currently fulfilled
		To indicate the benefits–side effects ratio one would find acceptable concerning new treatments
		To reveal through one's behaviours new/unmet therapeutic needs
	Representation‐relevant risks	Some therapeutic needs and preferences may be forgotten or downplayed
		Difficulty in communicating needs and preferences
		Self‐selection bias
Community knowledge	Components of community knowledge	Insights about the therapeutic needs and preferences of a community
		Insights about contextual factors that inform the therapeutic needs of a community
		Insights about the habits of a community: where members can be easily found, how they like to be approached
		Insights on differences and similarities in the therapeutic needs of potential subgroups of the community
	How to acquire community knowledge	Through analysis of quantitative data collected by the patient organisation
		Through analysis of qualitative data collected by the patient organisation
		Conducting surveys on social media
		Talking to community members
		Developing communication channels for community members to share their needs, preferences and other relevant insights
	Representation activities based on community knowledge	To explain statistics concerning patients' preferences
		To share insights about a community's therapeutic needs and preferences
		To ensure that a community's needs and preferences are taken into account by drug developer teams
	Representation‐relevant risks	Only some needs and preferences, experienced by subgroups of a community with whom one is familiar/by oneself are shared
		Distance between the representative and the community
		The same people acting as representatives
		Too time‐consuming to develop and keep up‐to‐date
Adaptive skills	Components of adaptive skills	Personality traits
		Communication/persuasive skills
		Ability to adjust to different environments
		Ability to correctly assess people and situations
		Ability to negotiate
	How to develop adaptive skills	Reading relevant materials
		Participating in training activities
		Taking part in engagement activities
	Application of adaptive skills	To successfully deploy one's knowledge
		To achieve one's/one's community/s goals
		To develop long‐term relationships with relevant stakeholders
	Representation‐relevant risks	Lack or limited involvement of people with relevant knowledge, but with limited adaptive skills
		Limited diversity of representatives
		Limited opportunities to practically train this set of skills
Sources of legitimacy	Diversity of representatives	Patient status of representatives (patients vs. nonpatients)
		At individual disease level versus broader disease category
		Gender of representatives
		Age of representatives
		Location of representatives
	Membership in a patient organisation	Member of a patient organisation
		Not (necessarily) a member of a patient organisation
	Knowledge of representatives	Autobiographical knowledge
		Drug development knowledge
		Community knowledge
	Experience of representatives	Previous experience representing patients
		No experience representing patients
	Links to other stakeholders	(lack of) links to the pharmaceutical industry
		Previous collaborations with the drug development teams
		Previous collaborations with (other) patient organisations in the same disease area
		Previous collaborations with (other) patient organisations in different disease areas
